# Supplementary material for: From sequence to enzyme mechanism using multi-label machine learning
Source: BMC Bioinformatics. 2014 May 19;15:150. doi: 10.1186/1471-2105-15-150 (PMC4229970; doi:10.1186/1471-2105-15-150)
Supplement: Additional file 2 — Java code of ml2db. Additional file ml2db_code.tar.gz contains the Java source code to run the multi-label machine learning experiments and save the results to database. The code’s Javadoc is included. [file 1471-2105-15-150-S2.zip › additional file 2/ml2db/utils/doc/uk/ac/standrews/utils/main/guiutils/GuiUtils.html]

GuiUtils


JavaScript is disabled on your browser.


- Overview
- Package
- Class
- Use
- Tree
- Deprecated
- Index
- Help

- Prev Class
- Next Class

- Frames
- No Frames

- All Classes

- Summary:
- Nested |
- Field |
- Constr |
- Method

- Detail:
- Field |
- Constr |
- Method


uk.ac.standrews.utils.main.guiutils

## Class GuiUtils

- java.lang.Object
- - java.awt.Component
  - - java.awt.Container
    - - java.awt.Window
      - - java.awt.Frame
        - - javax.swing.JFrame
          - - uk.ac.standrews.utils.main.guiutils.GuiUtils

- All Implemented Interfaces:
  :   java.awt.image.ImageObserver, java.awt.MenuContainer, java.io.Serializable, javax.accessibility.Accessible, javax.swing.RootPaneContainer, javax.swing.WindowConstants

  ---

    

  ```
  public class GuiUtils
  extends javax.swing.JFrame
  ```

  Gui utilities

  Version:
  :   10 Dec 2007

  Author:
  :   Luna De Ferrari luna.deferrari-at-ed.ac.uk

  See Also:
  :   Serialized Form

- - ### Nested Class Summary

    - ### Nested classes/interfaces inherited from class java.awt.Window

      `java.awt.Window.Type`
    - ### Nested classes/interfaces inherited from class java.awt.Component

      `java.awt.Component.BaselineResizeBehavior`
  - ### Field Summary

    - ### Fields inherited from class javax.swing.JFrame

      `EXIT_ON_CLOSE`
    - ### Fields inherited from class java.awt.Frame

      `CROSSHAIR_CURSOR, DEFAULT_CURSOR, E_RESIZE_CURSOR, HAND_CURSOR, ICONIFIED, MAXIMIZED_BOTH, MAXIMIZED_HORIZ, MAXIMIZED_VERT, MOVE_CURSOR, N_RESIZE_CURSOR, NE_RESIZE_CURSOR, NORMAL, NW_RESIZE_CURSOR, S_RESIZE_CURSOR, SE_RESIZE_CURSOR, SW_RESIZE_CURSOR, TEXT_CURSOR, W_RESIZE_CURSOR, WAIT_CURSOR`
    - ### Fields inherited from class java.awt.Component

      `BOTTOM_ALIGNMENT, CENTER_ALIGNMENT, LEFT_ALIGNMENT, RIGHT_ALIGNMENT, TOP_ALIGNMENT`
    - ### Fields inherited from interface javax.swing.WindowConstants

      `DISPOSE_ON_CLOSE, DO_NOTHING_ON_CLOSE, HIDE_ON_CLOSE`
    - ### Fields inherited from interface java.awt.image.ImageObserver

      `ABORT, ALLBITS, ERROR, FRAMEBITS, HEIGHT, PROPERTIES, SOMEBITS, WIDTH`
  - ### Constructor Summary

    Constructors

    Constructor and Description

    `GuiUtils()`
  - ### Method Summary

    Methods

    | Modifier and Type | Method and Description |
    | --- | --- |
    | `static void` | `errorPopUp(java.lang.String message)` Error pop-up window with message (icon: stop) |
    | `static java.io.File` | `fileChooserWithPreMessage(java.lang.String message, java.lang.String barTitle)` 1. |
    | `static void` | `interactiveError(boolean isInteractive, java.lang.String message)` Emits a popup if the mode is interactive or writes to System.err if non interactive |
    | `static void` | `interactiveMessage(boolean isInteractive, java.lang.String message)` Emits a popup if the mode is interactive or writes to System.out if non interactive |
    | `static void` | `main(java.lang.String[] args)` For testing |
    | `static void` | `messagePopUp(java.lang.String message, java.lang.String barTitle)` Pop-up window with message (icon: information > "i") |
    | `static int` | `multiOptionsPopUp(java.lang.String message, java.lang.String title, java.lang.String[] options)` A multiple options popup. |
    | `static java.io.File` | `simpleDirectoryChooser()` Returns the directory chosen by the user in a file chooser window. |
    | `static java.io.File` | `simpleFileChooser()` Returns the file chosen by the user in a file chooser window. |
    | `static java.lang.String` | `simpleInputDialog(java.lang.String question, java.lang.String title)` Creates a simple input pop-up |
    | `static void` | `simpleMessagePopUp(java.lang.String message)` Pop-up window with message (icon: information > "i") |
    | `static int` | `yesNoPopup(java.lang.String message, java.lang.String title, java.lang.String option1, java.lang.String option2)` A two options popup (usually for yes / no) The second option (usually the no) is pre-selected. |

    - ### Methods inherited from class javax.swing.JFrame

      `getAccessibleContext, getContentPane, getDefaultCloseOperation, getGlassPane, getGraphics, getJMenuBar, getLayeredPane, getRootPane, getTransferHandler, isDefaultLookAndFeelDecorated, remove, repaint, setContentPane, setDefaultCloseOperation, setDefaultLookAndFeelDecorated, setGlassPane, setIconImage, setJMenuBar, setLayeredPane, setLayout, setTransferHandler, update`
    - ### Methods inherited from class java.awt.Frame

      `addNotify, getCursorType, getExtendedState, getFrames, getIconImage, getMaximizedBounds, getMenuBar, getState, getTitle, isResizable, isUndecorated, remove, removeNotify, setBackground, setCursor, setExtendedState, setMaximizedBounds, setMenuBar, setOpacity, setResizable, setShape, setState, setTitle, setUndecorated`
    - ### Methods inherited from class java.awt.Window

      `addPropertyChangeListener, addPropertyChangeListener, addWindowFocusListener, addWindowListener, addWindowStateListener, applyResourceBundle, applyResourceBundle, createBufferStrategy, createBufferStrategy, dispose, getBackground, getBufferStrategy, getFocusableWindowState, getFocusCycleRootAncestor, getFocusOwner, getFocusTraversalKeys, getIconImages, getInputContext, getListeners, getLocale, getModalExclusionType, getMostRecentFocusOwner, getOpacity, getOwnedWindows, getOwner, getOwnerlessWindows, getShape, getToolkit, getType, getWarningString, getWindowFocusListeners, getWindowListeners, getWindows, getWindowStateListeners, hide, isActive, isAlwaysOnTop, isAlwaysOnTopSupported, isAutoRequestFocus, isFocusableWindow, isFocusCycleRoot, isFocused, isLocationByPlatform, isOpaque, isShowing, isValidateRoot, pack, paint, postEvent, removeWindowFocusListener, removeWindowListener, removeWindowStateListener, reshape, setAlwaysOnTop, setAutoRequestFocus, setBounds, setBounds, setCursor, setFocusableWindowState, setFocusCycleRoot, setIconImages, setLocation, setLocation, setLocationByPlatform, setLocationRelativeTo, setMinimumSize, setModalExclusionType, setSize, setSize, setType, setVisible, show, toBack, toFront`
    - ### Methods inherited from class java.awt.Container

      `add, add, add, add, add, addContainerListener, applyComponentOrientation, areFocusTraversalKeysSet, countComponents, deliverEvent, doLayout, findComponentAt, findComponentAt, getAlignmentX, getAlignmentY, getComponent, getComponentAt, getComponentAt, getComponentCount, getComponents, getComponentZOrder, getContainerListeners, getFocusTraversalPolicy, getInsets, getLayout, getMaximumSize, getMinimumSize, getMousePosition, getPreferredSize, insets, invalidate, isAncestorOf, isFocusCycleRoot, isFocusTraversalPolicyProvider, isFocusTraversalPolicySet, layout, list, list, locate, minimumSize, paintComponents, preferredSize, print, printComponents, remove, removeAll, removeContainerListener, setComponentZOrder, setFocusTraversalKeys, setFocusTraversalPolicy, setFocusTraversalPolicyProvider, setFont, transferFocusDownCycle, validate`
    - ### Methods inherited from class java.awt.Component

      `action, add, addComponentListener, addFocusListener, addHierarchyBoundsListener, addHierarchyListener, addInputMethodListener, addKeyListener, addMouseListener, addMouseMotionListener, addMouseWheelListener, bounds, checkImage, checkImage, contains, contains, createImage, createImage, createVolatileImage, createVolatileImage, disable, dispatchEvent, enable, enable, enableInputMethods, firePropertyChange, firePropertyChange, firePropertyChange, firePropertyChange, firePropertyChange, firePropertyChange, getBaseline, getBaselineResizeBehavior, getBounds, getBounds, getColorModel, getComponentListeners, getComponentOrientation, getCursor, getDropTarget, getFocusListeners, getFocusTraversalKeysEnabled, getFont, getFontMetrics, getForeground, getGraphicsConfiguration, getHeight, getHierarchyBoundsListeners, getHierarchyListeners, getIgnoreRepaint, getInputMethodListeners, getInputMethodRequests, getKeyListeners, getLocation, getLocation, getLocationOnScreen, getMouseListeners, getMouseMotionListeners, getMousePosition, getMouseWheelListeners, getName, getParent, getPeer, getPropertyChangeListeners, getPropertyChangeListeners, getSize, getSize, getTreeLock, getWidth, getX, getY, gotFocus, handleEvent, hasFocus, imageUpdate, inside, isBackgroundSet, isCursorSet, isDisplayable, isDoubleBuffered, isEnabled, isFocusable, isFocusOwner, isFocusTraversable, isFontSet, isForegroundSet, isLightweight, isMaximumSizeSet, isMinimumSizeSet, isPreferredSizeSet, isValid, isVisible, keyDown, keyUp, list, list, list, location, lostFocus, mouseDown, mouseDrag, mouseEnter, mouseExit, mouseMove, mouseUp, move, nextFocus, paintAll, prepareImage, prepareImage, printAll, removeComponentListener, removeFocusListener, removeHierarchyBoundsListener, removeHierarchyListener, removeInputMethodListener, removeKeyListener, removeMouseListener, removeMouseMotionListener, removeMouseWheelListener, removePropertyChangeListener, removePropertyChangeListener, repaint, repaint, repaint, requestFocus, requestFocusInWindow, resize, resize, revalidate, setComponentOrientation, setDropTarget, setEnabled, setFocusable, setFocusTraversalKeysEnabled, setForeground, setIgnoreRepaint, setLocale, setMaximumSize, setName, setPreferredSize, show, size, toString, transferFocus, transferFocusBackward, transferFocusUpCycle`
    - ### Methods inherited from class java.lang.Object

      `equals, getClass, hashCode, notify, notifyAll, wait, wait, wait`
    - ### Methods inherited from interface java.awt.MenuContainer

      `getFont, postEvent`

- - ### Constructor Detail


    - #### GuiUtils

      ```
      public GuiUtils()
      ```
  - ### Method Detail


    - #### errorPopUp

      ```
      public static void errorPopUp(java.lang.String message)
      ```

      Error pop-up window with message (icon: stop)

      Parameters:
      :   `message` -


    - #### fileChooserWithPreMessage

      ```
      public static java.io.File fileChooserWithPreMessage(java.lang.String message,
                                           java.lang.String barTitle)
      ```

      1. tells the user the reason she should select a file using a pop-up. 2.
      opens a file chooser windows. 3. Returns the file chosen by the user.

      Returns:
      :   the file chosen by the user


    - #### interactiveError

      ```
      public static void interactiveError(boolean isInteractive,
                          java.lang.String message)
      ```

      Emits a popup if the mode is interactive or writes to System.err if non
      interactive

      Parameters:
      :   `isInteractive` - true if the mode is interactive
      :   `message` - the error message


    - #### interactiveMessage

      ```
      public static void interactiveMessage(boolean isInteractive,
                            java.lang.String message)
      ```

      Emits a popup if the mode is interactive or writes to System.out if non
      interactive

      Parameters:
      :   `isInteractive` - true if the mode is interactive
      :   `message` - the message


    - #### main

      ```
      public static void main(java.lang.String[] args)
      ```

      For testing

      Parameters:
      :   `args` -


    - #### messagePopUp

      ```
      public static void messagePopUp(java.lang.String message,
                      java.lang.String barTitle)
      ```

      Pop-up window with message (icon: information > "i")

      Parameters:
      :   `message` - the message in the pop-up
      :   `barTitle` - the title for the bar above the pop-up


    - #### multiOptionsPopUp

      ```
      public static int multiOptionsPopUp(java.lang.String message,
                          java.lang.String title,
                          java.lang.String[] options)
      ```

      A multiple options popup. The first option is pre-selected.

      Parameters:
      :   `message` - the message / question
      :   `title` - the title above the popup window frame
      :   `options` - an array of string options

      Returns:
      :   the int representing the chosen option (0 = first option, 1=
          second option ... -1 = the window was closed without selecting)


    - #### simpleDirectoryChooser

      ```
      public static java.io.File simpleDirectoryChooser()
      ```

      Returns the directory chosen by the user in a file chooser window.

      Returns:
      :   the chosen directory (as File object)


    - #### simpleFileChooser

      ```
      public static java.io.File simpleFileChooser()
      ```

      Returns the file chosen by the user in a file chooser window.

      Returns:
      :   the file chosen


    - #### simpleInputDialog

      ```
      public static java.lang.String simpleInputDialog(java.lang.String question,
                                       java.lang.String title)
      ```

      Creates a simple input pop-up

      Parameters:
      :   `question` - the question
      :   `title` - the pop-up bar title

      Returns:
      :   the user input


    - #### simpleMessagePopUp

      ```
      public static void simpleMessagePopUp(java.lang.String message)
      ```

      Pop-up window with message (icon: information > "i")

      Parameters:
      :   `message` - the message in the pop-up


    - #### yesNoPopup

      ```
      public static int yesNoPopup(java.lang.String message,
                   java.lang.String title,
                   java.lang.String option1,
                   java.lang.String option2)
      ```

      A two options popup (usually for yes / no) The second option (usually the
      no) is pre-selected.

      Parameters:
      :   `message` - the message / question
      :   `title` - the title above the popup window frame
      :   `option1` - the option "To be..."
      :   `option2` - the other option "Not to be..."

      Returns:
      :   the int representing the chosen option (0 = first option, 1=
          second option ... -1 = the window was closed without selecting)


- Overview
- Package
- Class
- Use
- Tree
- Deprecated
- Index
- Help

- Prev Class
- Next Class

- Frames
- No Frames

- All Classes

- Summary:
- Nested |
- Field |
- Constr |
- Method

- Detail:
- Field |
- Constr |
- Method
